# Identification of mimotopes of *Mycobacterium leprae* as potential diagnostic reagents

**DOI:** 10.1186/1471-2334-13-42

**Published:** 2013-01-25

**Authors:** Silvana M Alban, Juliana Ferreira de Moura, João Carlos Minozzo, Marcelo Távora Mira, Vanete Thomaz Soccol

**Affiliations:** 1 Engenharia de Bioprocessos e Biotecnologia, Universidade Federal do Paraná, Curitiba 81531-990, Brasil; 2 Departamento de Patologia Básica, Universidade Federal do Paraná, Curitiba, 81531-990, Brazil; 3 Centro de Produção e Pesquisa de Imunobiológicos, Secretária de Saúde do Estado do Paraná, Piraquara, 83302-160, Brazil; 4 Centro de Ciências Biológicas e da Saúde, Pontifícia Universidade Católica do Paraná, Curitiba, 80215-901, Brazil

## Abstract

**Background:**

An early diagnostic test for detecting infection in leprosy is fundamental for reducing patients’ sequelae. The currently used lepromin is not adequate for disease diagnosis and, so far, no antigen to be used in intradermoreaction has proved to be sensitive and specific for that purpose. Aiming at identifying new reagents to be used in skin tests, candidate antigens were investigated.

**Methods:**

Random peptide phage display libraries were screened by using antibodies from leprosy patients in order to identify peptides as diagnostic reagents.

**Results:**

Seven different phage clones were identified using purified antibodies pooled from sera of leprosy patients. When the clones were tested with serum samples by ELISA, three of them, 5A, 6A and 1B, allowed detecting a larger number of leprosy patients when compared to controls. The corresponding peptides expressed by selected phage clones were chemically synthesized. A pilot study was undertaken to assess the use of peptides in skin tests. The intradermal challenge with peptides in animals previously sensitized with *Mycobacterium leprae* induced a delayed-type hypersensitivity with peptide 5A (2/5) and peptide 1B (1/5). In positive controls, there was a 3/5 reactivity for lepromin and a 4/5 reactivity of the sensitized animals with soluble extract of *M. leprae.*

**Conclusions:**

The preliminary data suggest that may be possible to develop reagents with diagnostic potential based on peptide mimotopes selected by phage display using polyclonal human antibodies.

## Background

Leprosy is a chronic granulomatous infectious disease caused by *M. leprae* that affects the skin and the peripheral nervous system [1]. This disease constitutes a public health problem in countries such as Brazil [2], where large numbers of patients with sequelae as a result of neural damages are still observed every year [3]. Upon the introduction of multidrug therapy in 1981 by the WHO, the prevalence of leprosy has been dramatically reduced, but over the last 5 years, more than 200,000 new cases have been detected each year [4]. These more recent data indicate that the transmission of leprosy still goes on [5], which makes it clear that new strategies - besides treatment - are necessary to eliminate the disease as a public health problem.

Leprosy presents a wide range of clinical manifestations determined by the immune response of the individual against the bacillus. Tuberculoid leprosy patients show a response that limits pathogen growth and result in few lesions containing rare (or absence of) bacilli however, nerve damage is often present. Lepromatous leprosy patients are susceptible to disseminated infection. Skin lesions are numerous and there is an uncontrolled proliferation of leprosy bacilli. Such clinical presentations correlate to the level of cell-mediated immunity (CMI) against *M. leprae*. Antibody responses are greater in lepromatous patients, indicating that humoral immunity does not contribute to host defense [6].

The delayed-type hypersensitivity reaction (DTH) in skin tests is considered a manifestation of the cell-mediated immunity [2]. In leprosy, cells of *M. leprae* from different origins and submitted to different processes of purification are the basis for different types of preparations employed in skin tests. Among those preparations, the most frequently used is lepromin, which corresponds to a suspension of heat-killed bacilli [7]. As *M. leprae* is not cultivated *in vitro*, cells for lepromin preparation can only be obtained from naturally infected human tissues (lepromas) or from experimentally infected armadillos [8].

Two types of positive skin reactions are described after the intradermal injection of 0.1 mL of lepromim: an early reaction (Fernandez Reaction), read after 48–72 hours; and a late reaction (Mitsuda Reaction), read after 3–4 weeks [9]. The Mitsuda reaction is measured as induration and provides a measure of the individual’s ability to mount a granulomatous response against mycobacterial antigens [7]. Positive Mitsuda reactions are seen in the vast majority of contacts and unexposed individuals, as well as in tuberculoid leprosy patients; in leprosy patients with lepromatous disease no response is observed. Therefore, reactivity to lepromin can not used for diagnosis, but has a prognostic value [9]. In Brazil, the *Centro de Produção e Pesquisa de Imunobiológicos* (CPPI) is the only supplier of lepromin antigen for the Brazilian Department of Health, and its production is based on lepromas obtained from lepromatous leprosy patients. This is a difficult process because the production of the antigen depends on access to rare lepromas that may be contaminated with other species of mycobacteria [10]. In addition, the strategy is associated with the risk of manipulation and inoculation of human biological material.

An alternative for replacing whole cells or purified fractions of *M. leprae* is to use synthetic peptides. Previous studies have demonstrated delayed-type hypersensitivity responses to peptides in skin tests [11,12]. Aiming at finding alternative diagnostic supplies for leprosy, peptides binding antibody from multibacillary leprosy patients were selected from phage displayed peptide libraries and assessed in their capacity to induce cell response in guinea-pigs sensitized with *M. leprae*.

## Methods

### Antigens

The *M. tuberculosis* H37Rv (ATCC 27294) strain was obtained from the *Instituto Adolfo Lutz*, São Paulo - Brazil. The cells were grown in Middlebrook 7H10 for 14–21 days at 37°C, followed by suspension in 0.9% NaCl and inactivation at 100°C for 15 minutes. Irradiated *M. leprae* whole cells isolated from armadillo liver were kindly supplied by Dr. J. S. Spencer from Colorado State University (Fort Collins-USA) through the National Institute of Allergy and Infectious Diseases/National Institutes of Health under contract N01-AI-25469. Bacilli in 0.9% NaCl containing 100 μg/mL phenylmethylsulfonyl fluoride (PMSF) and 2 mM ethylenediaminetetraacetic acid (EDTA) were broken by sonication (Sonopuls HD 2200, Bandelin, Berlin-Germany) four cycles of 15 minutes [13]. Following lysis, the soluble fraction was separated by centrifugation at 10000 × g for 20 minutes at 4°C, and the protein concentration was determined using the Quant-iT Protein Assay kit (Invitrogen, California-USA).

### Patient serum samples

Serum samples of 10 paucibacillary patients (PB), 23 multibacillary patients (MB), and 26 household contacts were obtained. The samples were collected at the *Hospital de Dermatologia Sanitária* (Piraquara, Paraná-Brazil), *Centro Regional de Especialidades-Barão* (Curitiba, Paraná-Brazil), *Fundação Pró-Hansen* (Curitiba, Paraná-Brazil), and at *Sociedade Filantrópica Humanitas* (São Jerônimo da Serra, Paraná-Brazil). Among the leprosy patients, 14 had not yet started the treatment by the time of blood collection, and 19 were under treatment (days - 4 months). Serum of 30 patients with pulmonary tuberculosis (TB) - under treatment (days - 3 months) - was obtained at the *Centro Regional de Especialidades-Barão* (Curitiba, Paraná-Brazil) and at the *Hospital Regional da Lapa ‘São Sebastião’* (Lapa, Paraná-Brazil). Leprosy and TB patients were diagnosed and classified by a specialized physician at the Health Units. Serum of 30 endemic controls was obtained from volunteers with no history of infection by leprosy and tuberculosis. In all cases, drawing of blood was carried out with informed consent and approved by the Research Ethics Committee of Federal University of Paraná (Comitê de Ética em Pesquisa, Setor de Ciências da Saúde, Universidade Federal do Paraná).

### Immunoglobulins anti-*M. leprae* and anti-*M. tuberculosis*

IgG of the sera pool from MB patients were obtained by precipitation with ammonium sulfate followed by chromatography using protein G-agarose [14]. The anti-*M. leprae* IgGs were recovered from immunoblots. For that, the *M. leprae* protein extract was resolved by SDS-PAGE in gradient polyacrylamide gel 10-20%; after having been transferred to the PVDF membrane, IgGs binding antigen immobilized in membrane were eluted with 0.1 M glycine, 0.15 M NaCl, pH 2.8 at room temperature for 30 minutes. Anti-*M. leprae* IgGs were dialyzed against PBS after neutralization with 1 M Tris–HCl, pH 9.0, and the protein concentration was determined by the Bradford method [15]. The same process was repeated using a sera pool from tuberculosis patients and *M. tuberculosis* protein in order to obtain anti-*M. tuberculosis* antibodies.

### Screening of random peptide phage display libraries

Linear (X15 and X8CX8) and constrained (XCX4CX and XCX8CX) random peptide libraries were obtained from J. Scott, Simon Fraser University (Burnaby BC-Canada). Peptides are expressed on the surface of protein pVIII of phage f88.4. Two panning strategies were used. In strategy I, the phages of libraries not bound to anti-*M. tuberculosis* immunoglobulins immobilized in immunotube were used in the first round of panning with anti-*M. leprae* immunoglobulins. In strategy II, the phages of libraries were directly used in the first round of panning with anti-*M. leprae* immunoglobulins. Panning was carried out according to the previously given description [16]. Briefly, one immunotube (Nunc, Roskilde-Denmark) was coated with anti-*M. leprae* immunoglobulin in 100 mM NaHCO_3_, pH 8.6 at 5 μg/1.5 mL and incubated overnight at 4°C. The immunotube was washed with TBST-0.05% (TBS Tween 20–0.05%), filled with blocking solution (TBST-0.05%, 3% BSA) and incubated for 2 hours at 37°C. For the first round of panning, 1.5 × 10^11^ phages of libraries X15 and XCX8CX, and 2.5 × 10^10^ phages of libraries X8CX8 and XCX4CX were incubated in TBST-0.05% with the immobilized antibodies. After the washings with TBST-0.5%, the bound phages were eluted with 1.5 mL 0.1 M glycine, pH 2.2, 1 mg/mL BSA. After neutralization with 2 M Tris–HCl, pH 9.0, the eluted phages were amplified by infecting a culture of *E. coli* K91. After incubation overnight at 37°C, the supernatant was obtained and precipitated with PEG/NaCl. In the remaining rounds, 2 × 10^11^ amplified phages of the previous cycle were incubated with IgG anti-*M. leprae*. The procedures were equal to those applied to the first round except for the fact that the concentration of the antibody immobilized to the immunotube was 1 μg and 0.5 μg in the second and subsequent rounds, respectively.

### Immunological screening of phage clones

After four rounds of panning, individual colonies containing phages were picked at random and grown overnight at 37°C in a 96-well microtiter plates in LB medium containing 20 μg/mL tetracycline. The plates were centrifuged (1600 × g, 20 minutes) and supernatant containing phages was analyzed regarding its binding to IgG from leprosy patients by ELISA. Microtiter plates (Falcon, BD Biosciences, California-USA) were coated by incubating 50 μL anti-phage antibody (Sigma-Aldrich, Missouri-USA) diluted 1:800 in 100 mM NaHCO_3_, pH 8.6, overnight at 4°C. The plates were washed with PBST-0.05% and blocked with 2% skimmed milk powder in PBST-0.05% for 1 hour at 37°C. The supernatant of each phage clone was added to each microtiter well. As a negative control, the supernatant of the *E. coli* K91 culture was used. After incubation for 2 hours at 37°C, the plate was washed and incubated with 100 μg/mL IgG for 1 hour at 37°C. After washing, peroxidase conjugated anti-human antibody (Sigma-Aldrich, Missouri-USA) - diluted 1:10000 in a blocking solution for 1 hour at 37°C - was added. After washing, bound antibodies were monitored for determining the peroxidase activity using ο-phenylenediamine dihydrochloride (OPD) (Sigma-Aldrich, Missouri-USA) as chromogen for measuring absorbance at 492 nm. The most reactive clones (absorbance at least twice as high as the negative control) were selected for DNA sequencing and subsequent identification of the peptide sequence inserted into the phages.

### Determination of peptide sequences

Single-stranded DNA was prepared out of phage clones using QIAprep Spin M13 kit (Qiagen, Hilden-Germany). Peptide sequences of the positive phage clones were determined by the BigDye Terminator v3.1 kit (Applied Biosystems, California-USA) using the reverse primer 5^′^-TCGGCAAGCTCTTTTAGG-3^′^.

### Phage-ELISA

Reactivity of the phage clones most reactive in the presence of human serum was measured by ELISA according to description given above. Microtiter plates were coated with anti-phage antibody and incubated with 2 × 10^10^ phages/mL followed by the addition of sera from leprosy patients, tuberculosis patients, household contacts of leprosy patients, and endemic controls, diluted 1:100. The detection of the reaction was performed with peroxidase conjugated anti-human antibody and OPD as chromogen.

### Synthetic peptides

Peptides were synthesized using solid phase 9-fluorenilmetoxycarbonyl (Fmoc) chemistry by PepTron (Daejon-Korea). Peptide purity was equal to or higher than 70%.

### Guinea pigs and delayed-type hypersensitivity responses

The experiments with these animals followed the guidelines of Brazilian College of Animal Experimentation (Colégio Brasileiro de Experimentação Animal - COBEA) and were approved by the institutional committee of Federal University of Paraná.

Three groups of five 250–300 g outbred female guinea pigs (*Cavia porcellus*) were used for the skin test. The antigens were diluted in 0.9% NaCl. The Mitsuda lepromin (4x10^7^ bacilli/mL) provided by the *Centro de Produção e Pesquisa de Imunobiológicos* (Piraquara, Paraná-Brazil) was used as positive control, and 0.9% NaCl was used as negative control. Five animals (group I) were sensitized by subcutaneous inoculation of 200 μg [17] of *M. leprae* in Freund’s incomplete adjuvant (Sigma-Aldrich, Missouri-USA). As control, five animals (group II) received 0.9% NaCl in Freund’s incomplete adjuvant subcutaneously. Control group III consisted of non-sensitized animals. After 30 days, each animal received, intradermally, 0.1 mL of the antigens: peptides (10 and 2 μg), peptide pool (10 and 2 μg), Mitsuda lepromin (positive control), soluble extract of *M. leprae* (10 μg, positive control) and 0.9% NaCl (negative control). The diameter of the skin reaction was measured after 24, 48, and 72 hours, 21 and 28 days.

### Bioinformatics

The peptide sequences were analyzed for sequence similarity using FASTA program on *M. leprae* sequence data (http://genolist.pasteur.fr/Leproma/).

## Results and discussion

**Figure 1:**
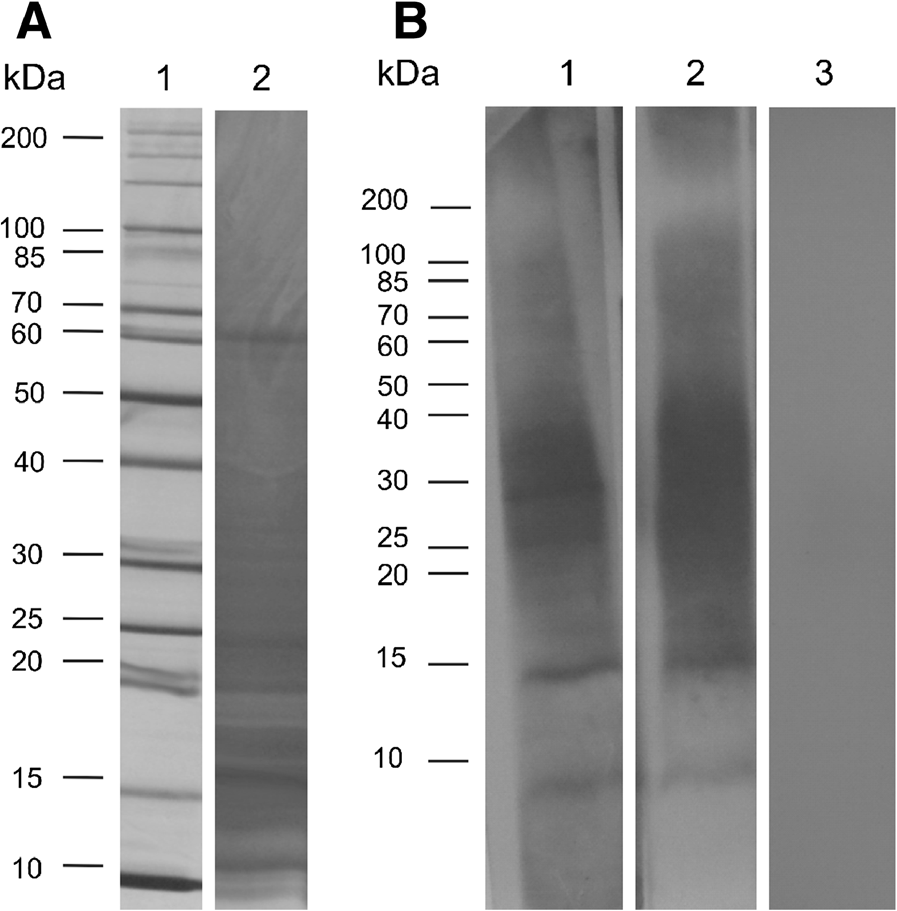
**SDS-PAGE and Western blot analysis of total extract of *M. leprae*.A**. Twenty micrograms of total extract of *M. leprae* were separated by 10-20% gradient SDS-PAGE and the gel was stained with silver nitrate (lane 2). Molecular weight marker is shown in the lane 1. **B**. The proteins were transferred to PVDF membrane and blots were probed with 1 μg/mL of MB patients’ total IgG (lane 1), 60 ng/mL of MB patients’ antigen-specific IgG (lane 2) and 1 μg/mL of PB patient’ total IgG (lane 3). Detection of the reaction was performed with anti-human IgG (Fc-specific)-peroxidase antibody (1:30000) and chemiluminescence.

In order to identify mimotopes of mycobacterial antigens, peptide libraries displayed by phage were selected by affinity using *M. leprae* antigen-specific antibodies. The antibodies were obtained from a pool of sera from MB patients. The IgGs were purified by ammonium sulfate precipitation and affinity chromatography followed by elution from membranes containing *M. leprae* antigens bound. The total IgGs from MB patients recognized proteins in *M. leprae* especially below 50 kDa (Figure 1B, lane 1). The same profile of reactivity was seen with antigen-specific IgGs (Figure 1B, lane 2), however, the antibody preparation method represent an important purification and concentration step, as relatively specific antibodies are purified from total IgG well as most of the extraneous IgGs active against other antigens are removed. Lipoarabinomannan (LAM) is a major antigen of *M. leprae* that is recognized by immunoglobulins in sera from leprosy patients [18] and runs as a broad diffuse band from 30–40 kDa as shown in Figure 1.

**Figure 2:**
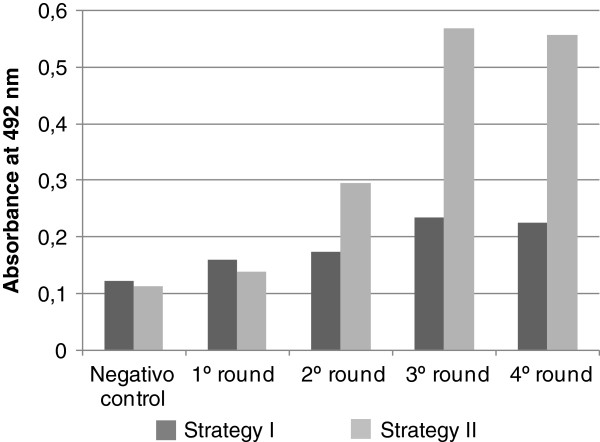
**Reactivity of phages pools selected by affinity strategies at each selection round by ELISA.** Microtiter plate was coated with anti-phage antibody (1:800) and incubated with 2 × 10^10^ phages/mL followed by the addition of IgG (100 μg/mL). The detection of the reaction was performed with peroxidase conjugated anti-human antibody and OPD as chromogen. Values of absorbance at 492 nm are means of duplicates. Wild phage was used as negative control.

Two panning strategies were used. In one of them, the phage pool with anti-*M. leprae* antibodies, was depleted of reactive phages with anti*-M. tuberculosis* antibodies. Four rounds of affinity selection were performed and the amplified phage pool of each selection round was assessed by ELISA (Figure 2). In both strategies, an increase of reactivity was observed with each round of panning. However, the best result was detected between the phages and the IgG obtained in strategy II.

The phage pool of the last selection round was then chosen to isolate individual positive clones. Each phage clone was assessed – by ELISA – regarding its ability to bind to IgG of multibacillary patients. In strategy I, 24 out of 400 clones analyzed showed recognition against patients’ antibodies, and 6 different peptide sequences were identified. In strategy II, 36 out of the 342 clones were recognized for binding to antibodies against leprosy and all expressed an identical sequence of 15 aminoacids.

**Figure 3:**
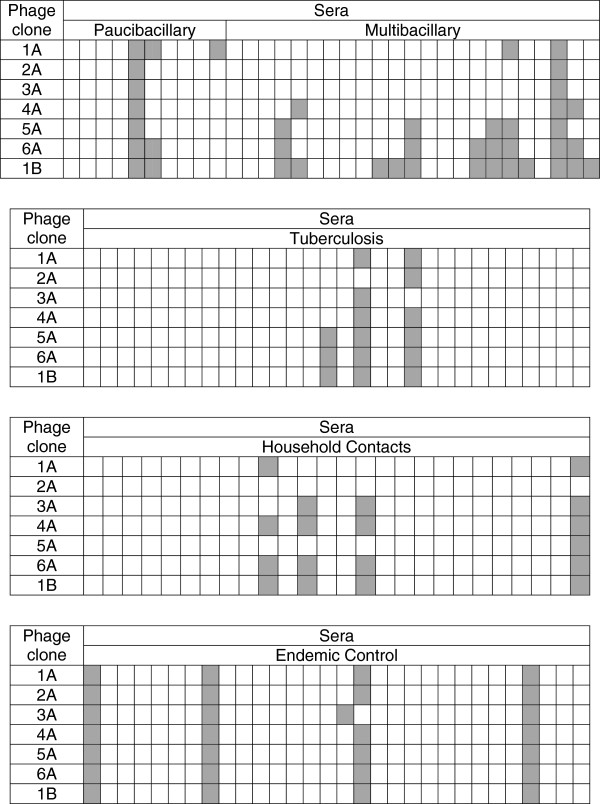
**Reactivity of phage clones by ELISA with sera of patients and controls.** Microtiter plates were coated with anti-phage antibody (1:800) and incubated with 2 × 10^10^ phages/mL followed by the addition of sera (1:100). The detection of the reaction was carried out with peroxidase conjugated anti-human antibody and OPD as chromogen. Each cell in the table represents an individual. The analysis was performed in duplicate and the hatched cells represent positive samples. The cut-off value was calculated as the average of the optical density plus twice the standard deviation obtained with endemic controls.

The respective phage clones carrying the identified peptide sequences were then assessed against sera of leprosy patients and controls by ELISA (Figure 3). Reactive control samples were the same regardless of the assessed clones, which could indicate an unspecific reaction between serum and phages.

Seven phage clones were selected and, among them, clones 5A, and 6A, clones isolated by strategy I, and 1B, clone isolated by strategy II, were detected in a higher number of leprosy patients when compared to the negative controls. Even for the selected clones, a low sensitivity was observed in the detection of leprosy patients. Each serum sample was also tested with wild phage by ELISA in order to evaluate the cross reaction of the serum with the phage without the insert. The results show that the reaction of the phage clones was stronger than that of the wild phage with the positive sera samples against the phage clones (data not shown), indicating that the reactivity happened exclusively between the antibodies and the peptides expressed on the surface of the phages.

The search for similarity of peptide sequences in Leproma database showed that the peptides selected mimic mainly proteins in *M. leprae* (Table 1). However, the LAM antigen, from which mimotopes can be isolated, would not be detected by FASTA approach. Further studies could reveal if those peptides represents mimotopes of the same antigen or of different antigens. Comparison of selected mimotopes and the native antigen sequence could lead to a better understanding of molecular mechanisms that participate in the immune response and the design of peptides for diagnostic purposes [19].

**Table 1:** **Similarity search between peptide sequence and *M. leprae *sequence database**

**Peptide**	**Size(amino acids)**	**Potential antigens**	
		***M. leprae *Sanger ID**	**Description**
5A	15	ML2028	Secreted antigen 85-B fbpB
		ML0781	Conserved hypothetical protein
		ML1135	Probable protoporhyrinogen oxidase hemK homolog
		ML0041	Possible secreted protease
		ML0053c	Possible conserved transmembrane protein
		ML2031	Conserved hypothetical protein
		ML0213	Possible cell cycle protein MesJ
		ML2307c	Probable transcriptional regulatory protein WhiB-like whiB4
		ML2137	Conserved hypothetical protein
		ML1177c	Probable conserved liproprotein lprD
6A	12	ML2263c	Probable naphthoate synthase menB
		ML2028	Secreted antigen 85-B fbpB
		ML2030	Probable resuscitation-promoting factor rpfC
		ML0053c	Possible conserved transmembrane protein
		ML0679	Hypothetical protein
		ML 0468c	Possible conserved integral membrane protein
		ML0213	Possible cell cycle protein MesJ
1B	15	ML2028	Secreted antigen 85-B fbpB
		ML0097	Secreted antigen 85-A FbpA
		ML2485c	Possible RNA methyltransferase
		ML1195	Isoleucyl-tRNA synthetase IleS
		ML2324	2-isopropylmalate synthase leuA
		ML2409c	Possible cytochrome C-type biogenesis protein ccsA
		ML2597	Probable conserved Mce associated protein
		ML0455c	Conserved hypothetical protein
		ML1037c	Conserved hypothetical protein
		ML0052c	Conserved hypothetical protein
		ML0130c	Probable methyltransferase
		ML0369c	Conserved hypothetical protein

Potential antigens corresponds to the best scores obtained for each peptide sequence as a query sequence against *M. leprae* database using FASTA search.

**Figure 4:**
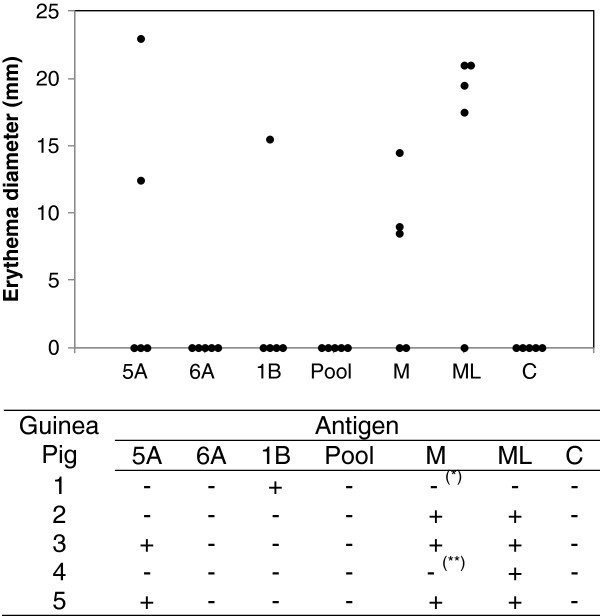
**Delayed-type hypersensitivity response induced by peptides in guinea-pigs.** Guinea-pigs were sensitized with 200 μg *M. leprae* in Freund’s incomplete adjuvant and - after 30 days - were inoculated intradermally with peptides and controls. Each group contained five animals. The result in 48 hours after application of 10 μg peptides, Mistsuda lepromin (M) with 4 × 10^6^ bacilli and 10 μg soluble extract of *M. leprae* (ML) is indicated. The peptide pool contain equimolar amounts of the three peptides. The diameter of the reaction corresponds to the average of horizontal and vertical diameters of the erythema. 0.9% NaCl (C) was used as negative control. * The animal responded after 72 hours. ** The animal responded after 21 days.

Since a humoral response depends on T helper cell response, the peptides identified were assessed regarding their ability to induce a delayed-type hypersensitivity response in guinea-pigs sensitized with *M. leprae*. The use of guinea-pigs constitutes the animal model used in delayed-type hypersensitivity tests with *M. leprae* antigens, according to description presented previously [17,20-22]. The intradermal challenge with peptides in animals previously sensitized with *M. leprae* induced delayed-type hypersensitivity. The maximum reading was observed in 48 hours and, therefore, that time was considered in the description of the results. No reactions were detected in animals sensitized with adjuvant as well as in non-sensitized animals. In the group sensitized with *M. leprae,* a DTH reaction was observed with peptide 5A (2/5), peptide 1B (1/5), Mitsuda lepromin (3/5), and with *M. leprae* (4/5) (Figure 4). For peptide 1B, the skin reaction was observed for both 2 and 10 μg doses with similar sizes - 16.5 mm and 15.5 mm respectively - whereas for peptide 5A, only the 10 μg dose generated a response. The two animals that did not respond to Mitsuda lepromin in 48 hours produced a response after 72 hours (induration) or 21 days (induration) after the application of the antigen. The detection of response with Mitsuda lepromin and the soluble extract of *M. leprae* indicate that the animals were sensitized. The skin reactions observed were erythematous; only one of the animals immunized with *M. leprae*, besides the erythema, also developed a papule as a result of applying peptide 5A.

The lack of cellular response in other animals is probably due to the influence of genetic factors involving genes MHC in the responses to peptides [11,23]. Thus, the mixture of peptides able to recognize different forms of disease or different genetic aspects could help to overcome problems of genetic restriction to individual peptides. Other factors that need to be evaluated are the dosage and the constitution of the antigen as, for example, the conjugation with proteins or modification of the peptide structure. Differences were found in the DTH response regarding the dosage of the inoculated peptide [11,24]. Other studies have tested peptides in the form of peptide-protein conjugation proteins [25] or as peptides modified by the addition of fatty acids and acetylation [26].

Differences in reactivity between phage clones and the corresponding free peptide have been described previously [27,28]. When in phage, the sequence could to assume a favorable position to act as binder of antibodies [29] and therefore this conformation could disappear when used as synthetic peptides. Likewise, the conformation of the peptide expressed on the phage surface may discourage recognition by antibodies. Thus, other peptide sequences identified but not included in DTH tests will be evaluated in future studies. The decision was to limit the tests to the phage clones that were reactive with larger number of patients. The selection of mimotopes was based on seroreactivity and therefore these mimetic peptides correspond to B cell epitopes. The strategy of the study was to test whether these peptides would be able to act as T cell epitopes. Serological and cellular assays, as proposed initiated this study may determine whether a peptide can comprise a serological or T cell test reagent. Tests for humoral immune response benefit lepromatous patients while tests for evaluation of cellular response will aid in the diagnosis of tuberculoid patients.

Lepromin is probably the only skin test antigen that reflects the individual’s ability to generate a granulomatous response to mycobacterial antigens, as opposed to the 48–72 hours delayed-type hypersensitivity response found with tuberculin and other skin tests [7]. In the lepromin test, the Fernandez reaction is similar to the reaction produced by tuberculin, since it assesses the delayed-type hypersentivity of the individual to *M. leprae* soluble antigens [22]. In this aspect, the present study proposes the use of peptides in skin tests to assess the cellular response, similar to the one captured by the Mantoux test used in tuberculosis. Besides *in vivo* studies, the peptides identified can be assessed in the future regarding their ability to induce INF-γ production by peripheral blood mononuclear cells of patients, aiming to verify whether those reagents allow the detection of the paucibacillary disease. However, the skin tests are more feasible and useful as compared to INF-γ production assays, since the latter is dependent on the access to advanced lab facilities and trained personnel, which limits its use in countries with limited resources [30], a proposal advocated in the present study. Recently, the development of a user-friendly assay to detect multiple cytokines [31] which can make INF-γ detection assays more accessible and easier to perform was reported.

Synthetic peptides prove to be very attractive to be used in diagnostic tests or for monitoring the degree of exposure to leprosy within different communities, or even an adjunct to leprosy control programs [32], since they are easy to produce with reproducibility, as well as high quality and purity control [33]. In addition, synthetic peptides can be easily obtained at a low production cost through chemical synthesis. Comparatively, crude antigens originating from *M. leprae* are complex mixtures that carry proteins of humans or animal tissues in their composition, which may pose biological risks.

## Conclusions

Peptides mimotopes were isolated from phage displayed libraries by screening purified antibodies derived from leprosy patients’ sera. Our strategy was to evaluate the ability of mimotopes to induce cellular response in DTH tests. The selected sets of peptides are also a starting point to the future design of more effective reagents for diagnostic purposes.

## Competing interests

The authors declare that they have no competing interests.

## Authors’ contributions

SMA participated in designing the study, carrying out the experiments, analyzing data and writing the manuscript. JFM conceived the study, participated in designing the study and writing the manuscript. JCM participated in the execution of experiment. MTM participated in analyzing the data and writing the manuscript. VTS conceived the study, participated in designing the study, data analysis and writing of the manuscript. All the authors read and approved this final manuscript.

## Pre-publication history

The pre-publication history for this paper can be accessed here:

http://www.biomedcentral.com/1471-2334/13/42/prepub
